# A Research Investigation into the Impact of Reinforcement Distribution and Blast Distance on the Blast Resilience of Reinforced Concrete Slabs

**DOI:** 10.3390/ma16114068

**Published:** 2023-05-30

**Authors:** Yangyong Wu, Jianhui Wang, Fei Liu, Chaomin Mu, Ming Xia, Shaokang Yang

**Affiliations:** 1 Institute of Defense Engineering, Academy of Military Sciences, People’s Liberation Army, Beijing 100850, China; yywu_aust@163.com (Y.W.);; 2 School of Safety Science and Engineering, Anhui University of Science and Technology, Huainan 232001, China; chmmu@mail.ustc.edu.cn

**Keywords:** reinforced concrete slabs, blast resistance, reinforcement distribution, blast distance, anti-blast performance, model tests

## Abstract

Reinforcement is one of the important factors affecting the anti-blast performance of reinforced concrete (RC) slabs. In order to study the impact of different reinforcement distribution and different blast distances on the anti-blast performance of RC slabs, 16 model tests were carried out for RC slab members with the same reinforcement ratio but different reinforcement distribution and the same proportional blast distance but different blast distances. By comparing the failure patterns of RC slabs and the sensor test data, the impact of reinforcement distribution and blast distance on the dynamic response of RC slabs was analyzed. The results show that, under contact explosion and non-contact explosion, the damage degree of single-layer reinforced slabs is more serious than that of double-layer reinforced slabs. When the scale distance is the same, with the increase of distance, the damage degree of single-layer reinforced slabs and double-layer reinforced slabs increases first and then decreases, and the peak displacement, rebound displacement and residual deformation near the center of the bottom of RC slabs gradually increase. When the blast distance is small, the peak displacement of single-layer reinforced slabs is smaller than that of double-layer reinforced slabs. When the blast distance is large, the peak displacement of double-layer reinforced slabs is smaller than that of single-layer reinforced slabs. No matter how large the blast distance, the rebound peak displacement of the double-layer reinforced slabs is smaller, and the residual displacement is larger. The research in this paper provides a reference for the anti-explosion design, construction and protection of RC slabs.

## 1. Introduction

In today’s world, peace and development have become the main theme of the times, and the world is in a relatively peaceful and stable situation. However, violent terrorist attacks and local wars caused by religious and racial discrimination and hegemonism have emerged in an endless stream, posing serious challenges to the safety protection design of buildings and structures around the world [1,2,3]. In addition, in the process of industrial production and processing, the use of various flammable and explosive dangerous goods is inevitable, which will bring potential risks to the safety of buildings and structures and seriously threaten the safety of people’s lives and property [4,5]. The explosion load is ignored in the design and construction of traditional buildings. Attention should be paid to the anti-explosion design of important buildings such as stations, schools, business centers and other places with dense traffic. It is necessary to perform a good job in the prevention of violent terrorist attacks [6,7,8].

The basic structural elements of a building include beams, slabs, columns and walls. Studying the impact of blast load on individual structural elements is of great significance [9,10,11,12]. When studying the overall building, various structural elements are coupled with each other, and it is difficult to distinguish the contribution of a certain structural element to the blast resistance of the building.

Slabs are important load-bearing components in buildings, so it is important to study the anti-explosion performance of slabs for the design and protection of buildings [13,14]. Traditional research is mainly based on typical RC slabs. By changing the reinforcement ratio [15,16,17,18,19,20,21,22,23,24,25], strength of the concrete [16,17,18], span of the slab [16], thickness of the slab [17,19,20], strength of the reinforcement [18] and scale distance [20,21], the data on the failure form, blast pit, displacement and reflected overpressure of the reinforced concrete slab after the explosion are obtained. The damage degree and anti-explosion performance of the RC slab are evaluated by the bearing angle [18], displacement [22], residual bearing capacity [23] and P-I curve [16,24]. Among them, the reinforcement ratio is the most influential factor.

Recent research on slabs mainly includes the application of new materials, reinforcement technology and the introduction of new failure prediction methods. In terms of new materials, on the one hand, RC slabs are made of carbon-fiber-reinforced polymer [26], low ductility reinforcement [27] and basalt-fiber-reinforced plastic bars [28], so the loss of slabs under an explosion load is smaller.

On the other hand, by changing the material of concrete, the RC slabs are made of ultra-high-strength concrete [29], ultra-high-performance fiber-reinforced concrete [30], superabsorbent polymer honeycomb concrete [31], 200 MPa ultra-high-performance fiber-reinforced concrete [32] and ultra-high-ductility concrete mixed with ultra-high-performance concrete [33,34], which have a better anti-explosion performance than ordinary RC slabs.

In terms of reinforcement technology, Mendonca et al. [35] studied the use of foam to strengthen RC slabs through experiments and concluded that the slabs strengthened with foam had different pressure modes compared with ordinary slabs, and that the displacement and acceleration increased instead. Maadoun et al. [26] strengthened the RC slab by bonding carbon-fiber-reinforced polymer (CFRP) and concluded that the strengthened slab has a better flexural bearing capacity and stiffness under an explosive load. Gao et al. [36] verified the finite element model of the ultra-high-performance concrete slab strengthened with polyurea based on the experiment. Through changing the reinforcement ratio and scale distance to carry out an anti-explosion numerical simulation of the slab, they obtained the prediction formula of the end rotation angle of the ultra-high-performance concrete slab strengthened with polyurea under a near-field explosion. Gao et al. [37] carried out an explosion resistance experiment of an RC slab with a porous energy-absorbing material foam aluminum protective layer, verified the finite element model based on the experimental data, studied the damage rule of the foam aluminum density and longitudinal reinforcement ratio on reinforced concrete and concluded that the greater the reinforcement ratio, the better the explosion resistance effect of the RC slab. Thiagarajan and Reynolds [38] studied the anti-explosion performance of high-strength concrete slabs strengthened with high-strength vanadium steel through an explosion simulator, concluded that slabs with a larger spacing of steel bars have a smaller ductility and gave the damage mode of the panel.

In terms of introducing new failure prediction methods, Almustafa et al. [39] studied the influence of 10 input characteristics on the maximum displacement of RC slabs under an explosive load based on the random forest algorithm. This method has achieved good results in predicting the maximum displacement of RC slabs, and is more efficient and accurate than the existing numerical calculation methods. Shishegaran et al. [40] evaluated various models based on normalized square error and fractional deviation and concluded that the best model for predicting the maximum deflection of the plate is multiple Ln equation regression.

In the latest research, both the application of new materials and the reinforcement of slabs will increase the construction cost of buildings. In previous studies, increasing the reinforcement ratio can enhance the anti-explosion performance of RC slabs, which will also lead to increased costs. In this paper, by fixing the reinforcement ratio and changing the distribution of reinforcement in the slabs, the difference in anti-explosion performance between single-layer reinforced slabs and double-layer reinforced slabs with the same reinforcement ratio under contact explosion and non-contact explosion was studied. This can determine which type of reinforcement distribution in slabs has a better blast resistance without increasing costs. In previous studies, the conditions for changing the scale distance to change the blast load were discussed. By using a fixed scale distance, the influence of blast distance on the blast resistance of RC slabs was studied in this paper, which can verify whether the load conditions determined by scale distance are reliable.

## 2. Test Overview

### 2.1. Design of Specimens

**Figure 1:**
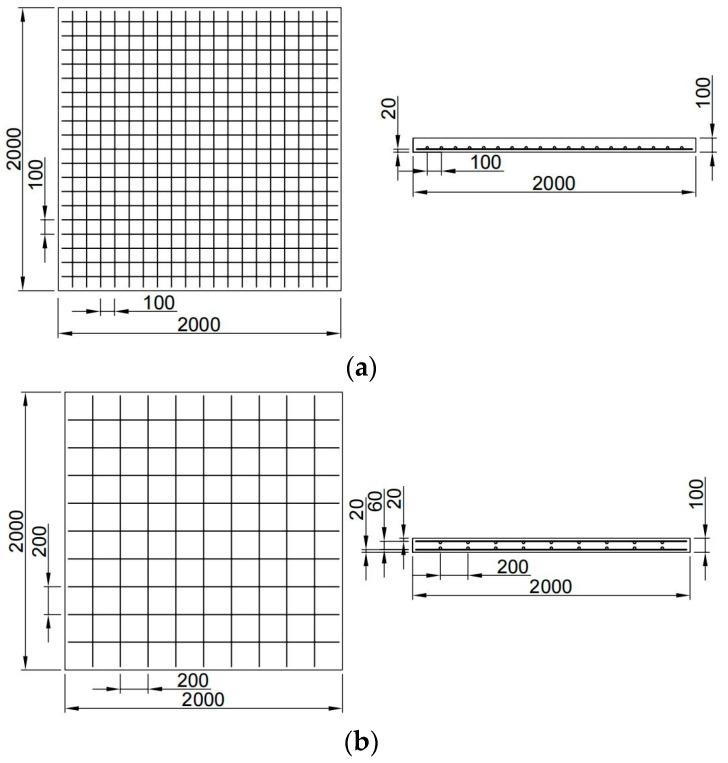
Reinforcement diagram of RC slabs; unit is mm. (**a**) Single-layer reinforced slab; (**b**) double-layer reinforced slab.

The size of slabs was 2000 mm × 2000 mm × 100 mm, and they were HRB400E-reinforced and had a diameter of 8 mm. The concrete strength grade was C40, and the thickness of concrete protective layer was 20 mm. Single-layer two-way reinforcement and double-layer two-way reinforcement were adopted for the components. The spacing of single-layer reinforcement slabs was 100 mm, the spacing of double-layer two-way reinforcement was 200 mm, and the spacing of layers was 600 mm. The number of single-layer reinforced slabs was S1–S8, and the number of double-layer reinforced slabs was D1–D8. The information of RC slab specimens is shown in Table 1, and the reinforcement diagram is shown in Figure 1.

### 2.2. Test Conditions

The working conditions of contact explosion are shown in Table 2, and the working conditions of non-contact explosion are shown in Table 3.

### 2.3. Material Properties

#### 2.3.1. Material Properties of Concrete

The concrete specimens were poured at the same time. Six concrete cubes with a size of 150 mm × 150 mm× 150 mm were retained for compression test when pouring the specimens. They were cured in the same environment as the components. The compressive strength of the six concrete cubes was 45.8, 48.2, 47.4, 46.8, 46.4 and 47.6 MPa, respectively, and the average compressive strength of the cubes was 47.0 MPa.

#### 2.3.2. Material Properties of Reinforcement

The model of reinforcement was HRB400E, and the diameter was 8 mm. Its mechanical properties are shown in Table 4.

### 2.4. Arrangement of Test

**Figure 2:**
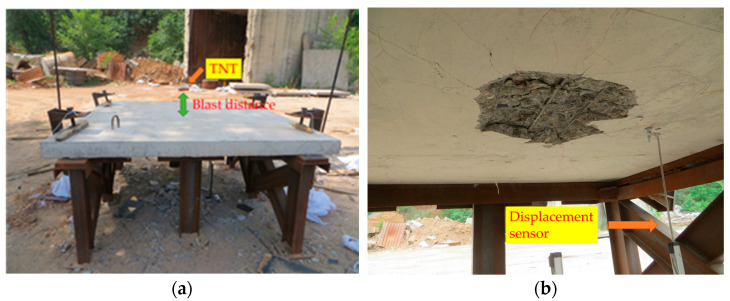
Experimental layout diagram. (**a**) Overall layout of the experiment; (**b**) arrangement of displacement sensor.

The sample of reinforced concrete slab was fixed onto a rigid frame made of I-beam, which adopts one-way support. Two clamps were installed on each side of the slab through bolt fastening. The contact explosion test installs explosives in the center of the slab, and the non-contact explosion test lifts explosives directly above the center of the slab. The explosives used in the test were stacked by standard TNT explosive blocks. The mass of standard TNT explosive block is 200 g, and the structural dimension is 100 mm × 50 mm × 25 mm, detonated with digital detonator. A displacement sensor with model DH5G107 was arranged in the center of bottom face to measure the dynamic displacement of the mid-span of the slab. Due to the fact that the concrete at the center of the bottom face may fall due to collapse, placing the displacement sensor here will damage. Therefore, move the displacement sensor RC board side by 30 cm. The experimental layout is shown in Figure 2.

## 3. Test Results and Analysis of Contact Explosion

### 3.1. Damage Form of Contact Explosion

**Figure 3:**
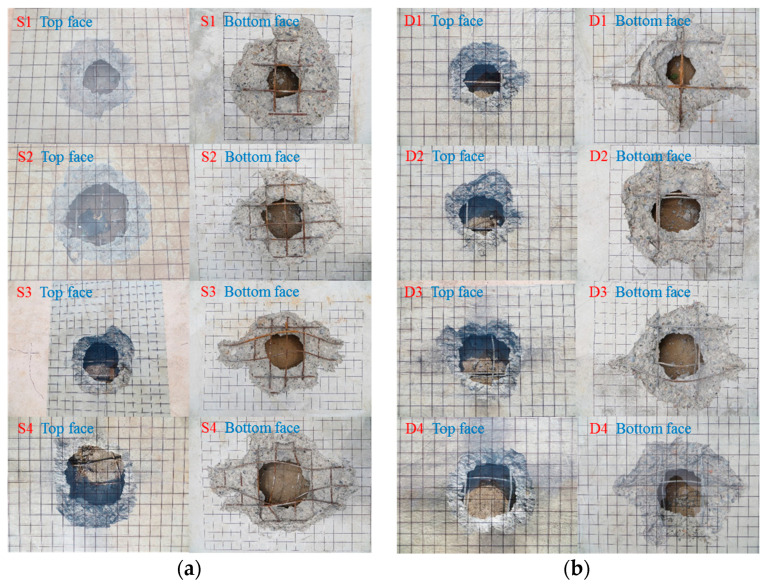
Contact explosion failure mode of RC slabs. (**a**) Single-layer reinforced slab; (**b**) Double-layer reinforced slab.

Under the contact explosion, the dynamic response law of the RC slab was studied by changing the charge. The contact explosion experimental results are shown in Table 5 and Figure 3.

### 3.2. Failure Mode of Contact Explosion

The failure mode of the RC slab under the contact explosion load is mainly local failure, which can be summarized into four types, namely explosion pit, explosion collapse, explosion penetration and explosion punching. The schematic diagram of the four failure modes is shown in Figure 3. Within the range of charge in this paper, the single-layer reinforced slab and double-layer reinforced slab both show explosive penetration damage, and the concrete medium near the center of the top face is crushed and peeled off to form a blast hole. The compression stress wave caused by the explosion will be reflected on the bottom face, and the resulting reflection stretching effect will cause the bottom face concrete to crack and collapse, thus forming a collapse hole. In addition, the top face blast hole and bottom face collapse hole will penetrate up and down.

### 3.3. Analysis of Damage Area of Contact Explosion

The damage area of the top face, the damage area of the bottom face and the diameter of the through hole of RC slabs under contact explosion are shown in Table 6.

Under the contact explosion, the top face is compressed and destroyed to form a blasting pit. The parameters that affect the blasting damage form of the top face include the explosion source factors (such as charge and explosive density) and the medium factors (such as concrete strength, concrete density, reinforcement strength, reinforcement density and wave velocity). Due to the main compression failure of the top face, it is assumed that the damage area of top face *A*_1_ is a function of charge *M*, the density of explosive *ρ*_1_, compressive strength of concrete *f_c_*, density of concrete *ρ*_2_, compressive strength of reinforcement *f_c_′*, density of reinforcement *ρ*_3_, wave velocity *V* and thickness of slabs *H*. It is assumed that the damage area of bottom face *A*_2_ is a function of charge *M*, density of explosive *ρ*_1_, tensile strength of concrete *f_t_*, density of concrete *ρ*_2_, tensile strength of reinforcement *f_t_′*, density of reinforcement *ρ*_3_, wave velocity *V* and thickness of slabs *H*. It is assumed that the through hole diameter *D* is a function of charge *M*, density of explosive *ρ_1_*, compressive strength of concrete *f_c_*, tensile strength of concrete *f_t_*, density of concrete *ρ*_2_, compressive strength of reinforcement *f_c_′*, tensile strength of reinforcement *f_t_′*, density of reinforcement *ρ*_2_, wave velocity *V* and thickness of slabs *H*. The dimension of each parameter is shown in Table 7. For the dimensional analysis of the damage area of top face *A*_1_, it can be expressed as Equation (1):

*A*_1_ = *f* (*M*, *ρ*_1_, *f_c_*, *ρ*_2_, *f_c_′*, *ρ*_3_, *V*, *H*) (1)

Select *M*, *f_c_*, *ρ*_2_ as independent variables and list three dimensionless Π values as shown in Equation (2):

(2) $$\prod_{1}=\frac{\rho_{1}}{M^{a_{1}}f_{c}{}^{a_{2}}\rho_{2}{}^{a_{3}}},\;\prod_{2}=\frac{f_{c}{}^{\prime }}{M^{b_{1}}f_{c}{}^{b_{2}}\rho_{2}{}^{b_{3}}},\;\prod_{3}=\frac{\rho_{3}}{M^{c_{1}}f_{c}{}^{c_{2}}\rho_{2}{}^{c_{3}}},\;\prod_{4}=\frac{V}{M^{d_{1}}f_{c}{}^{d_{2}}\rho_{2}{}^{d_{3}}},\;\prod_{5}=\frac{A_{1}}{M^{e_{1}}f_{c}{}^{e_{2}}\rho_{2}{}^{e_{3}}},\;\prod_{6}=\frac{H}{M^{f_{1}}f_{c}{}^{f_{2}}\rho_{2}{}^{f_{3}}}$$

The expression of each dimensionless quantity Π is shown in Equation (3):

(3) $$ML^{-3}=M^{a_{1}}{\left(ML^{-1}T^{-2}\right)}^{a_{2}}{\left(ML^{-3}\right)}^{a_{3}}\;ML^{-1}T^{-2}=M^{b_{1}}{\left(ML^{-1}T^{-2}\right)}^{b_{2}}{\left(ML^{-3}\right)}^{b_{3}}\;ML^{-3}=M^{c_{1}}{\left(ML^{-1}T^{-2}\right)}^{c_{2}}{\left(ML^{-3}\right)}^{c_{3}}\;LT^{-1}=M^{d_{1}}{\left(ML^{-1}T^{-2}\right)}^{d_{2}}{\left(ML^{-3}\right)}^{d_{3}}\;L^{2}=M^{e_{1}}{\left(ML^{-1}T^{-2}\right)}^{e_{2}}{\left(ML^{-3}\right)}^{e_{3}}\;L=M^{f_{1}}{\left(ML^{-1}T^{-2}\right)}^{f_{2}}{\left(ML^{-3}\right)}^{f_{3}}$$

Determine the indexes of Π according to the principle of dimensional consistency as shown in Equation (4):

(4) $$\prod_{1}=\frac{\rho_{1}}{M^{0}f_{c}{}^{0}\rho_{2}{}^{1}},\;\prod_{2}=\frac{f_{c}{}^{\prime }}{M^{0}f_{c}{}^{1}\rho_{2}{}^{0}},\;\prod_{3}=\frac{\rho_{3}}{M^{0}f_{c}{}^{0}\rho_{2}{}^{1}},\;\prod_{4}=\frac{V}{M^{0}f_{c}{}^{1/2}\rho_{2}{}^{1/2}},\;\prod_{5}=\frac{A_{1}}{M^{2/3}f_{c}{}^{0}\rho_{2}{}^{2/3}},\;\prod_{6}=\frac{H}{M^{1/3}f_{c}{}^{0}\rho_{2}{}^{-1/3}}$$

The dimensional function relationship can be obtained as shown in Equations (5) and (6):

(5) $$\frac{A_{1}}{M^{2/3}\rho_{2}{}^{2/3}}=f(\frac{\rho_{1}}{\rho_{2}},\frac{f_{c}{}^{\prime }}{f_{c}},\frac{\rho_{3}}{\rho_{2}},\frac{V}{f_{c}{}^{1/2}\rho_{2}{}^{1/2}},\frac{H}{M^{1/3}\rho_{2}{}^{-1/3}})$$

(6) $$A_{1}=M^{2/3}\rho_{2}{}^{2/3}f(\frac{\rho_{1}}{\rho_{2}},\frac{f_{c}{}^{\prime }}{f_{c}},\frac{\rho_{3}}{\rho_{2}},\frac{V}{f_{c}{}^{1/2}\rho_{2}{}^{1/2}},\frac{H}{M^{1/3}\rho_{2}{}^{-1/3}})$$

The explosion and the material of media in the experiment are constant and the density of explosive *ρ*_1_, compressive strength of concrete *f_c_*, density of concrete *ρ*_2_, compressive strength of reinforcement *f_c_′*, density of reinforcement *ρ*_3_, wave velocity *V* and thickness of slabs *H* are constant, so the above function form can be simplified as Equation (7):

*A*_1_ = *k*_1_*M*^2/3^ + *a* (7)

In the same way, the functional form of the damage area of the bottom face can be simplified as Equation (8):

*A*_2_ = *k*_2_*M*^2/3^ + *b* (8)

The functional form of the diameter of through hole can be simplified as Equation (9):

*D* = *k*_3_*M*^1/3^ + *c* (9)

**Figure 4:**
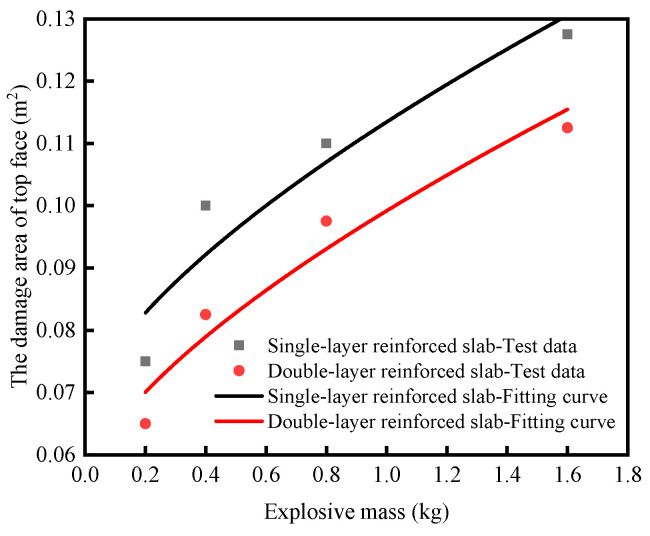
The change rule of the damage area of top face under contact explosion with the charge.

**Figure 5:**
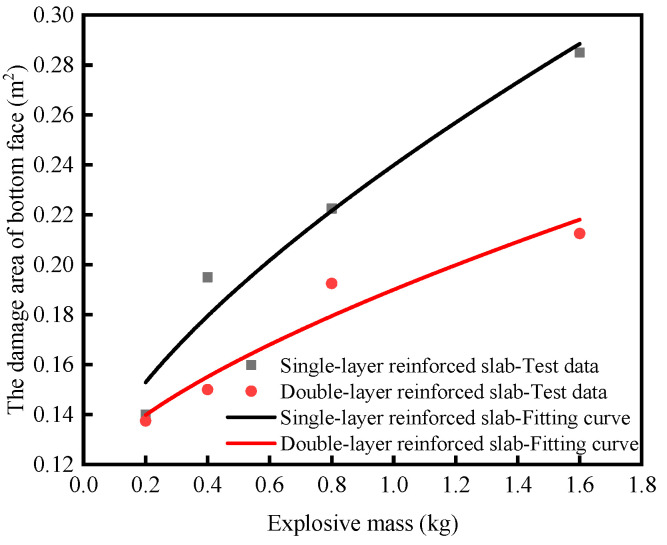
The change rule of the damage area of bottom face under contact explosion with the charge.

**Figure 6:**
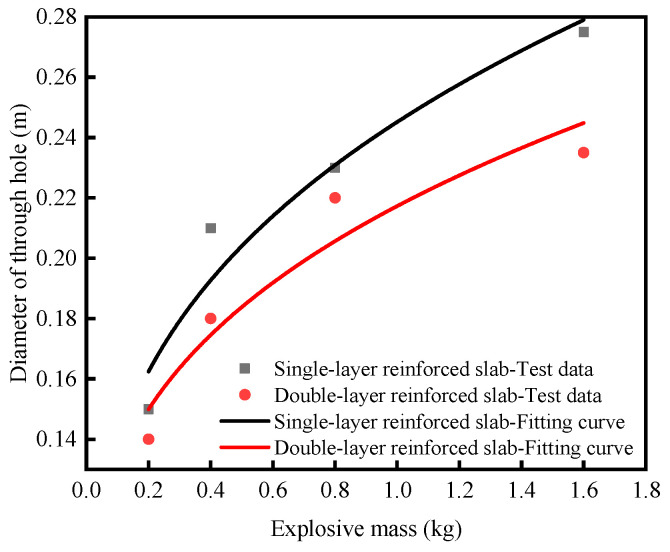
The change rule of the diameter of through hole under contact explosion with the charge.

Based on the test data of single-layer reinforced slabs and double-layer reinforced slabs, the fitting formula of the damage area of the top face *A*_1_, the damage area of the bottom face *A*_2_ and the diameter of through hole *D* are obtained by fitting the function form derived from dimensional analysis, as shown in Equations (10)–(15). The fitting curve is shown in Figure 4, Figure 5 and Figure 6, and the determination coefficient *R*^2^ of the fitting curve is greater than 0.8. For the explosion test, the fitting result is ideal. It can be seen from the formula that *k*_1_, *k*_2_ and *k*_3_ are parameters reflecting the anti-explosion performance of the medium. The smaller their values, the better the anti-explosion performance of the RC slab.

Single-layer reinforced slabs: *A*_1_ = 0.047*M*^2/3^ + 0.067    *R*^2^ = 0.902 (10)

Double-layer reinforced slabs: *A*_1_ = 0.044*M*^2/3^ + 0.055    *R*^2^ = 0.919 (11)

Single-layer reinforced slabs: *A*_2_ = 0.132*M*^2/3^ + 0.108    *R*^2^ = 0.942 (12)

Double-layer reinforced slabs: *A*_2_ = 0.076*M*^2/3^ + 0.114    *R*^2^ = 0.906 (13)

Single-layer reinforced slabs: *D =* 0.199*M*^1/3^ + 0.046    *R*^2^ = 0.913 (14)

Double-layer reinforced slabs: *D =* 0.162*M*^1/3^ + 0.055    *R*^2^ = 0.882 (15)

## 4. Experimental Results and Analysis of Non-Contact Explosion

### 4.1. Damage Form of Non-Contact Explosion

**Figure 7:**
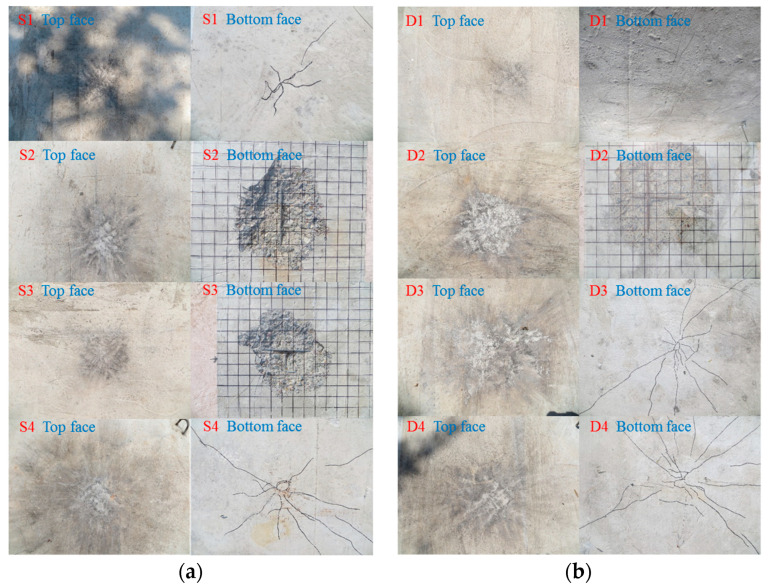
Non-contact explosion failure mode of RC slabs. (**a**) Single-layer reinforced slab; (**b**) double-layer reinforced slab.

Under the non-contact explosion, the dynamic response law of the RC slab is studied by changing the charge. The contact explosion experimental results are shown in Table 8 and Figure 7.

### 4.2. Failure Mode of Contact Explosion

The failure modes of RC slabs under non-contact explosive loads are mainly local failure and overall bending failure. The explosion shock wave causes damage on the blast face of the slab, and there is no crack or fine crack on the blast face. When the compression stress wave propagates to the bottom face of the slab, it will reflect and transmit, and the compression stress wave will be transformed into a tensile wave. Because of the low tensile strength of the concrete, the center of the bottom face will crack due to bending, the concrete will crack or even peel off and the slab will be bent.

### 4.3. Analysis of Damage Area of Contact Explosion

The damage area of the bottom face is shown in Table 9.

It can be seen from Figure 7 and Table 9 that the damage of the single-layer reinforced slab is more serious than that of the double-layer reinforced slab. When the scale distance is constant, with an increase in the blast distance, there will be slight cracks on the top face of the slab. The blast face of the slab will gradually increase from only cracks to concrete falling off, and then the concrete falling off area will gradually decrease until only cracks appear. The severity of the damage form will first increase and then decrease. This is because the initial burst distance and charge quantity are relatively small. Due to the size effect, it is difficult for the smaller-size explosives to damage the larger-size reinforced concrete slab. With an increase in the blast distance and charge quantity, the shackles of the size effect are broken, and the explosive produces large local damage on the reinforced concrete slab, but the damage at this time is caused by the joint action of the blast load and explosive gas after the explosion. With a further increase in the blast distance and charge quantity, the damage caused by explosives on the reinforced concrete slab gradually changes from local damage to overall damage. Due to the increase in blast distance, the explosive gas after the explosion of explosives escapes into the air. At this time, only the blast load acts on the reinforced concrete slab, resulting in a gradual reduction in the damage caused by explosives on the reinforced concrete slab.

### 4.4. Response Analysis of Displacement

**Figure 8:**
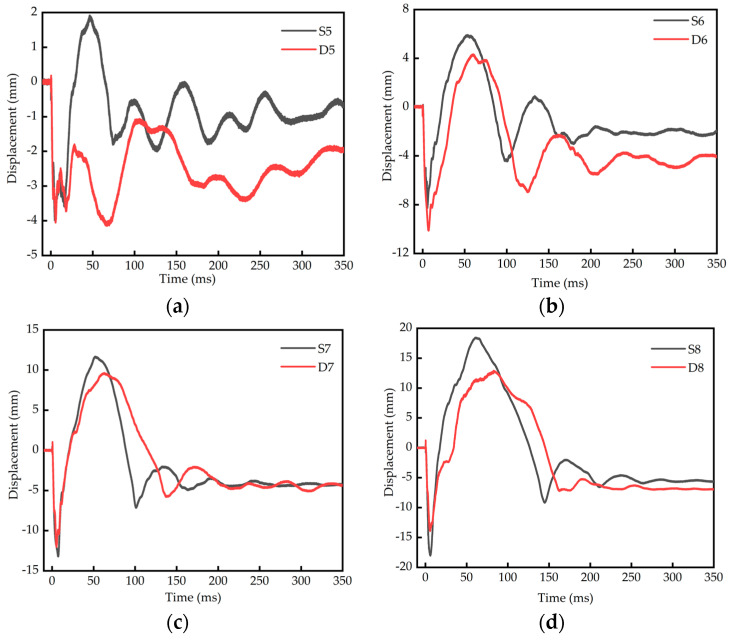
Comparison of displacement–time-history curves of single-layer and double-layer RC slabs. (**a**) 0.2 kg; (**b**) 0.4 kg; (**c**) 0.8 kg; (**d**) 1.6 kg.

The comparison of displacement–time-history curves of single-layer reinforced slabs and double-layer reinforced slabs is shown in Figure 8. The displacement data of the test are shown in Table 10.

It can be seen from Figure 8 and Table 10 that the peak displacement of a single-layer reinforced plate is 3.99 mm and 8.24 mm, respectively, the peak displacement of a double-layer reinforced slab is 4.10 mm and 10.10 mm, respectively, and the peak displacement of a single-layer reinforced slab is smaller when the blast distance and charge are relatively small; that is, the blast distance is 0.25 m and 0.32 m and the charge is 0.2 kg and 0.4 kg. When the blast distance and charge are relatively large—that is, when the blast distance is 0.40 m and 0.50 m and the charge is 0.8 kg and 1.6 kg—the displacement peak of the double-layer reinforced slab is 11.98 mm and 13.91 mm, respectively, the displacement peak of the single-layer reinforced slab is 13.19 mm and 17.99 mm, respectively, and the displacement peak of the double-layer reinforced slab is smaller. When the blast distance and charge are relatively small, the RC slab is mainly subject to local damage. At this time, the reinforcement of the single-layer reinforced slab is closer to the bottom face, and its displacement peak value is smaller. When the blast distance and charge are relatively large, the RC slab is mainly subject to overall damage. At this time, the overall structure of the double-layer reinforced slab is better, and its displacement peak value is smaller. The peak rebound displacement of a single-layer reinforced slab is 1.85, 5.87, 11.64 and 18.40 mm, respectively, and that of a double-layer reinforced slab is −1.10, 4.28, 9.59 and 13.12 mm, respectively. The peak rebound displacement of a single-layer reinforced slab is greater than that of a double-layer reinforced slab. The residual deformation of a single-layer reinforced slab is 0.70, 1.99, 4.25 and 5.61 mm, respectively, and that of a double-layer reinforced slab is 1.97, 4.06, 4.36 and 6.93 mm, respectively. The residual deformation of a single-layer reinforced slab is less than that of a double-layer reinforced slab.

**Figure 9:**
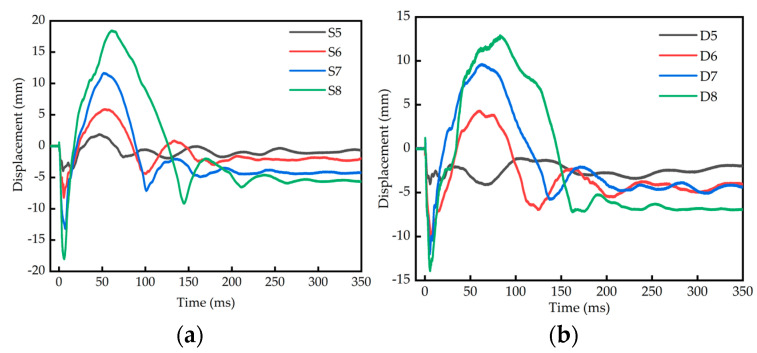
Comparison of displacement–time-history curves of RC slabs with different blasting distances. (**a**) Single-layer reinforced slab; (**b**) double-layer reinforced slab.

The comparison of displacement–time-history curves of RC slabs with different blast distances is shown in Figure 9. It can be seen from Figure 9 that the peak displacement, rebound peak displacement and residual deformation of both single-layer and double-layer reinforced slabs increase with an increase in the blast distance and charge.

## 5. Discussion

In this paper, the damage degree of RC slabs can be determined through the failure patterns and test data. From the perspective of failure patterns, the degree of damage to reinforced concrete slabs increases with an increase in charge under contact explosion, and, under the same conditions, the damage degree of double-layer reinforced slabs is significantly smaller than that of single-layer reinforced slabs. The damage degree of RC slabs increases with an increase in charging under a non-contact explosion, and, under the same conditions, the damage degree of double-layer reinforced slabs is significantly smaller than that of single-layer reinforced slabs.

However, from the perspective of test data, the results of the test are not entirely consistent. The damage of RC slabs is often related to the dynamic response of the RC slabs. The parameter tested in this paper was displacement, and the maximum bearing rotation angle $\theta_{max}$ can be calculated through the peak displacement. The dimensionless parameter $\theta_{max}$ is a standard for evaluating the degree of damage to reinforced concrete slab components. The calculation formula for the maximum bearing rotation angle $\theta_{max}$ is shown in Equation (16) [41]:

(16) $$\theta_{max}=\tan^{-1}\left(\frac{x_{\max}}{L/2}\right)$$

where *L* is the span of the RC slabs and $x_{\max}$ is the maximum displacement at the mid-span of the RC slabs.

In ref. [41], it is pointed out that the larger the maximum bearing rotation angle $\theta_{max}$, the greater the degree of damage to the RC slabs. For RC slabs of the same size, the larger the peak displacement, the greater the degree of damage to the RC slabs. From the test data, it can be seen that, with an increase in the blast distance and charge, the peak displacement gradually increases, which means that the degree of damage to the RC slabs increases. When the blast distance and charge are small, the displacement of the double-layer reinforced slabs is greater than that of the single-layer reinforced slabs, which means that the damage degree of the double-layer reinforced slabs is greater than that of the single-layer reinforced slabs. When the blast distance and charge are large, the displacement of the single-layer reinforced slabs is greater than that of the double-layer reinforced slabs, which means that the damage degree of the single-layer reinforced slabs is greater than that of the double-layer reinforced slabs. This is not entirely consistent with the results obtained based on the failure patterns. When the degree of damage determined by different standards is inconsistent, it is necessary to comprehensively consider the degree of damage obtained by various standards. Usually, the standard with the most severe degree of damage can be used as the criterion.

Liu et al. [42] conducted experiments on arch structure with the same scale distance (0.5 m·kg^−1/3^) and different blast distances and found that, the larger the blast distance, the more severe the damage to the arch structure and the larger the peak displacement. However, in this article, an inconsistent conclusion was drawn, where, the larger the blast distance, the greater the failure of the slab, which first increases and then decreases, but the peak displacement always increases. The difference in scale distance between the two is not significant, and the reason for different conclusions may be due to the different range of blast distance. In the experiment of the former, the blast distance range was 0.5–1.0 m, whereas, in the experiment of this paper, the blast distance range was 0.25–0.50 m.

## 6. Conclusions

In this paper, field chemical explosion experiments were carried out on RC slabs with the same reinforcement ratio but different reinforcement distribution and the same blast distance but different scale distance. The influence of different reinforcement distribution and different blast distance on the anti-blast performance of RC slabs was studied and the damage form and test data of RC slabs were compared and analyzed.

1. The charge increased from 0.2 kg to 1.6 kg under contact explosion, and the damage of single-layer reinforced slabs was more serious than that of double-layer reinforced slabs. The fitting relationships of two RC slabs between the damage area of the top face, the damage area of the bottom face, the diameter of the through hole and charge were obtained. The damage area of the top face of single-layer reinforced slabs was 12.8–21.2% larger than that of double-layer reinforced slabs. The damage area of the bottom face of single-layer reinforced slabs was 1.8–34.1% larger than that of double-layer reinforced slabs. The diameter of the through hole of single-layer reinforced slabs was 4.5–17.0% larger than that of double-layer reinforced slabs.
2. When the scale distance was the same, the blast distance increased from 0.25 m to 0.50 m under non-contact explosion, the damage degree of single-layer reinforced slabs and double-layer reinforced slabs increased first and then decreased and the peak of displacement, the peak of rebound displacement and residual deformation near the center of the bottom face gradually increased.
3. When the blast distance was small, the peak displacement of single-layer reinforced slabs was 2.8–22.6% larger than that of double-layer reinforced slabs. When the blast distance was large, the peak displacement of double-layer reinforced slabs was 10.1–29.3% larger than that of single-layer reinforced slabs. No matter how the blast distance changed, the peak rebound displacement of the double-layer reinforced slabs was 17.6–27.1% smaller than that of the single-layer reinforced slabs, and the residual deformation of the double-layer reinforced slabs was 1.03–2.81 times that of the single-layer reinforced slabs.
4. When designing and constructing RC slabs, double-layer or multi-layer reinforcement can be considered to improve the blast resistance of RC slabs. When studying blast load conditions, not only scale distance but also blast distance should be considered.

## Figures and Tables

**Table 1:** Information of RC slab specimens.

Types of RC Slabs	Model of Reinforcement	Model of Concrete	Reinforcement Ratio (%)	Number
Single-layer reinforced slab	HRB400E	C40	0.45	8
Double-layer reinforced slab	HRB400E	C40	0.45	8

**Table 2:** Working conditions of contact explosion.

Types of RC Slabs	Specimen	Charge (kg)
Single-layer reinforced slab	S1	0.2
	S2	0.4
	S3	0.8
	S4	1.6
Double-layer reinforced slab	D1	0.2
	D2	0.4
	D3	0.8
	D4	1.6

**Table 3:** Working conditions of non-contact explosion.

Types of RC Slabs	Specimen	Charge (kg)	Blast Distance (m)	Scale Distance (m·kg^−1/3^)
Single-layer reinforced slab	S1	0.2	0.25	0.43
	S2	0.4	0.32	0.43
	S3	0.8	0.40	0.43
	S4	1.6	0.50	0.43
Double-layer reinforced slab	D1	0.2	0.25	0.43
	D2	0.4	0.32	0.43
	D3	0.8	0.40	0.43
	D4	1.6	0.50	0.43

**Table 4:** Mechanical properties of reinforcement.

Model of Reinforcement	Elastic Modulus (GPa)	Yield Strength (MPa)	Tensile Strength (MPa)	Yield Strain (%)	Elongation (%)
HRB400E	200	455	587.5	0.23	21

**Table 5:** Test results of contact explosion of RC slabs.

Types of RC Slabs	Specimen	Description of Phenomenon
Single-layer reinforced slabs	S1	The reinforced concrete slab has a through hole, with a hole diameter of 15 cm. Four bars are exposed transversely and two bars are exposed longitudinally. The bending value of the bars is 2 cm.
	S2	The reinforced concrete slab has a through hole, with a hole diameter of 21 cm. Five bars are exposed horizontally, four bars are exposed longitudinally and the bending value of the bars is 5 cm.
	S3	The reinforced concrete slab has a through hole, with a hole diameter of 23 cm. Five bars are exposed horizontally and longitudinally, and the bending value of the bars is 10 cm.
	S4	The reinforced concrete slab has a through hole, with a hole diameter of 27.5 cm. Five bars are exposed transversely and seven bars are exposed longitudinally. The bending value of the bars is 10.5 cm.
Double-layer reinforced slabs	D1	The reinforced concrete slab has a through hole, with a hole diameter of 14 cm, and two bars are exposed horizontally and longitudinally. The bending value of the first layer of reinforcement is 3 cm, and the bending value of the second layer of reinforcement is 4 cm.
	D2	The reinforced concrete slab has a through hole, with a hole diameter of 18 cm. Two bars are exposed horizontally and longitudinally. The bending value of the first layer of reinforcement is 4 cm, and the bending value of the second layer of reinforcement is 6 cm.
	D3	The reinforced concrete slab has a through hole, with a hole diameter of 22 cm. Two bars are exposed horizontally and longitudinally. The bending value of the first layer of reinforcement is 6.5 cm, and the bending value of the second layer of reinforcement is 10.5 cm.
	D4	The reinforced concrete slab has a through hole, with a hole diameter of 23.5 cm. Two bars are exposed horizontally and longitudinally. The bending value of the first layer of reinforcement is 7.5 cm, and the bending value of the second layer of reinforcement is 11.5 cm.

**Table 6:** Measurement data of damage form under contact explosion.

Types of RC Slabs	Specimen	Charge (kg)	The Damage Area of Top Face (cm^2^)	The Damage Area of Bottom Face (cm^2^)	Diameter of Through Hole (cm)
Single-layer reinforced slabs	S1	0.2	750	1400	15
	S2	0.4	1000	1950	21
	S3	0.8	1100	2225	23
	S4	1.6	1275	2850	27.5
Double-layer reinforced slabs	D1	0.2	650	1375	14
	D2	0.4	825	1500	18
	D3	0.8	975	1925	22
	D4	1.6	1125	2125	23.5

**Table 7:** Dimension of blasting parameters.

Parameter	Symbol	Dimensions
Charge	*M*	M
Density of explosive	*ρ*_1_	ML^−3^
Compressive strength of concrete	*f_c_*	ML^−1^T^−2^
Tensile strength of concrete	*f_t_*	ML^−1^T^−2^
Density of concrete	*ρ*_2_	ML^−3^
Compressive strength of reinforcement	*f_c_′*	ML^−1^T^−2^
Tensile strength of reinforcement	*f_t_′*	ML^−1^T^−2^
Density of reinforcement	*ρ*_3_	ML^−3^
Wave velocity	*V*	LT^−1^
The damage area of top face	*A*_1_	L^2^
The damage area of bottom face	*A*_2_	L^2^
The diameter of through hole	*D*	L
Thickness of slabs	*H*	L

**Table 8:** Test results of non-contact explosion of RC slabs.

Types of RC Slabs	Specimen	Description of Phenomenon
Single-layer reinforced slabs	S5	There are traces of explosion on the top face, but no crater or crack is formed; there is a small crack in the center of the bottom face.
	S6	There are traces of explosion on the top face, but there are no explosion pits and tiny cracks; there is a seismic collapse pit on the bottom face with an area of 1075 cm^2^ and a depth of 4.5 cm. Three bars are exposed horizontally and one bar is exposed longitudinally.
	S7	There are traces of explosion on the top face, but there are no explosion pits and tiny cracks; there is a collapse pit on the bottom face with an area of 975 cm^2^ and a depth of 4 cm. Two bars are exposed horizontally and two bars are exposed longitudinally.
	S8	There are traces of explosion on the top face, but there are no explosion pits and tiny cracks; there are circumferential cracks and cracks emanating from the center to the periphery on the bottom face, and the diameter of circumferential cracks is 7 cm.
Double-layer reinforced slabs	D5	The reinforced concrete slab is free of damage and cracks.
	D6	There are traces of explosion on the top face, but there are no explosion pits and tiny cracks; there is a seismic collapse pit on the bottom face with an area of 1400 cm^2^ and a depth of 3.5 cm. One steel bar is exposed horizontally and two steel bars are exposed longitudinally.
	D7	There are traces of explosion on the top face, but there are no explosion pits and tiny cracks; there are circumferential cracks and cracks emanating from the center to the periphery on the bottom face, and the diameter of circumferential cracks is 6 cm.
	D8	There are traces of explosion on the top face, but there are no explosion pits and tiny cracks; there are circumferential cracks and cracks emanating from the center to the periphery on the bottom face, and the diameter of circumferential cracks is 5 cm.

**Table 9:** Measurement data of damage form under non-contact explosion.

Types of RC Slabs	Specimen	Explosive Mass (kg)	Blast Distance(m)	Scale Distance (m·kg^−1/3^)	The Damage Area of the Bottom Face (cm^2^)
Single-layer reinforced slab	S5	0.2	0.25	0.43	-
	S6	0.4	0.32	0.43	1075
	S7	0.8	0.40	0.43	975
	S8	1.6	0.50	0.43	-
Double-layer reinforced slab	D5	0.2	0.25	0.43	-
	D6	0.4	0.32	0.43	1400
	D7	0.8	0.40	0.43	-
	D8	1.6	0.50	0.43	-

**Table 10:** The displacement data of test.

Specimen	The Peak of Displacement (mm)	The Peak of Rebound Displacement (mm)	Residual Deformation (mm)
S5	−3.99	1.85	−0.70
S6	−8.24	5.87	−1.99
S7	−13.19	11.64	−4.25
S8	−17.99	18.40	−5.61
D5	−4.10	−1.10	−1.97
D6	−10.10	4.28	−4.06
D7	−11.98	9.59	−4.36
D8	−13.91	13.12	−6.95

## Data Availability

The data presented in this study are available on request from the corresponding author.
